# Implementation Patterns and Perceived Value of the SEXIT Method in School Health Care

**DOI:** 10.1111/jocn.70135

**Published:** 2025-10-13

**Authors:** Per Andreas Persson, Kristina Areskoug Josefsson, Malin Lindroth, Sofia Hammarström

**Affiliations:** ^1^ Region Västra Götaland Knowledge Centre for Sexual Health Gothenburg Sweden; ^2^ Department of Health Sciences University West Trollhättan Sweden; ^3^ Department of Behavioural Sciences, Faculty for Health Sciences Oslo Metropolitan University Oslo Norway

**Keywords:** adolescents, health promotion, school health services, sexual health, violence

## Abstract

**Aim:**

To explore implementation patterns and perceived value of the SEXIT (SEXual health Identification Tool) method in the school health care (SHC) setting in Sweden.

**Design:**

Mixed method survey using an online questionnaire with closed and free‐text response options.

**Methods:**

115 SHC professionals who had completed SEXIT training responded to an online questionnaire (response rate 26%), between March and May 2024. Closed questions were answered on a five‐point Likert scale, and responses trichotomised. Quantitative data were analysed using descriptive statistics, qualitative data with a deductive qualitative content analysis. CROSS guideline was used.

**Results:**

70 of 115 SHC professionals used SEXIT in their work. Findings suggest that SEXIT is appropriate and useful, supporting communication about topics such as sexual health and violence that both pupils and professionals may avoid addressing. Implementation patterns showed that 61% of those who had completed training also used SEXIT. 63% of those used SEXIT during regular health dialogues, but some did not use it with all pupils. The perceived value was that most SHC professionals felt that SEXIT helped them get a better understanding of the pupil's situation, an objection that it was too time‐consuming.

**Conclusion:**

Most SHC professionals who had completed SEXIT training used the method regularly and perceived the method as valuable and facilitating discussions about sexual ill health and experiences of violence with pupils. There are indications that SHC services fail to identify particularly at‐risk young people. Sexual health inequity persists, as some structurally marginalised and vulnerable youths are excluded from the SEXIT dialogues. A follow‐up study will focus on pupils' experiences.

**Relevance:**

This study validates SEXIT in a new setting, SHC, and is relevant for the promotion of sexual and reproductive health for all, and for preventing violence and sexual ill health among young people.

**Summary**

- What does this paper contribute to the wider global clinical community?
  ○ The results may be of use for school health care professionals and school health care leaders as they could serve as a basis for understanding the value of using tools that facilitate communication about sexual health and violence with youths in the school health care context.
  ○ This paper points out the strengths of SEXIT but also the pitfalls that professionals may encounter when working with the method. SEXIT should be used for all pupils to ensure the inclusion of structurally marginalised and vulnerable groups.

## 1 Introduction

Sexual ill health, including sexually transmitted infections (STIs), unintended pregnancy and sexual violence, is a serious public health concern among adolescents (Starrs et al. 2018). Adolescence is a developmental period during which individuals are vulnerable to experiencing sexual concerns (Slater and Robinson 2014). This is not surprising given the adolescent developmental characteristics, including sexual maturation, markedly increased sexual activity, proneness to risk‐taking (or chance‐taking) and peer pressure (Cacciatore et al. 2019; Forsyth and Rogstad 2015; Lindroth and Löfgren‐Mårtenson 2013).

Schools can improve sexual and reproductive health for young people, and school health services have a key role in this form of health promotion (UNICEF, WHO and UNESCO 2024; Wellings et al. 2006). Promoting sexual and reproductive health can involve screening for and detecting sexual ill health and experiences of violence (Hammarström 2021). In this work, the school nurse has an important role to play in advocating and promoting evidence‐based health services, which can help prevent STIs and unwanted pregnancies (Beech and Sayer 2018). It is crucial that health services detect ill health to avoid further, and perhaps even lifelong, suffering for the individual (and potential partners) and to avoid unnecessary costs for society (Santaularia et al. 2014). Today, however, many adolescents who suffer from sexual ill health remain undetected by health services (Sully et al. 2020).

In Sweden, an interview study with school nurses suggested that school nurses often used locally or regionally designed structured screening tools for psychosocial health (Silivri et al. 2023). However, there were few if any screening tools that included aspects related to sexual and reproductive health and rights (SRHR). The questions in the questionnaires used were described as too general, which required the school nurses to ask follow‐up questions, and they expressed a wish for a structured health questionnaire adapted for pupils from varied cultural backgrounds that could guide and support their ability to address and support SRHR (Silivri et al. 2023).

SEXual health Identification Tool (SEXIT) is an evidence‐based assessment tool developed for use at Swedish youth clinics and is intended to facilitate dialogue and professionals' identification of young people exposed to, or at risk of, sexual ill health and violence (Hammarström 2021). SEXIT was developed in response to needs expressed by clinical professionals who lacked a useful tool for working with young people, and it consists of education (a 1‐day main session and 1‐h booster sessions) for professionals, a 22‐item structured questionnaire for the young person, and a manual for professionals (Hammarström 2021). The SEXIT questionnaire is available in Swedish, Arabic, Dari, Somali, Tigrinya, English and in easy‐to‐read Swedish with explanatory pictures. According to the SEXIT method, all youths are offered the opportunity to answer the SEXIT questions as a routine, and they can choose to accept or decline (Hammarström et al. 2022). A systematic use of the SEXIT method facilitates the identification of young people exposed to, or at risk of, sexual ill health and violence by increasing the consistency and quality of assessments at the youth clinics (Hammarström 2021). School health care (SHC) in Sweden has also started to incorporate SEXIT in its statutory health dialogues, but this use of SEXIT has not been fully evaluated. School nurses find questionnaires useful when covering different aspects of health in the health dialogues (Kostenius 2021), which implies that SEXIT could be a valuable tool in SHC. Implementing a new method involves learning, and professionals must know what to do (Billett 2016). Successful work with SEXIT involves applying the method in a uniform manner; for example, all pupils should be offered the opportunity to answer the questions to prevent certain young people from being singled out (Hammarström et al. 2022). This study therefore focuses on implementation patterns of SEXIT in the SHC setting, such as frequency of use, adaptation of the model, collegial support, and how SHC professionals perceive the value of SEXIT in their health promotion work.

## 2 Background

### 2.1 SRHR in Young People

In Sweden, young people aged 16 to 24 are the most vulnerable group in society regarding being exposed to violence, and there are sub‐groups who are at higher risk (The Swedish National Council for Crime Prevention 2021). Adolescent girls and young women are more likely than adolescent boys and young men to be victims of sexual and domestic violence, and young LGBTQI people are more exposed to violence than heterosexual youths. Young people with disabilities are more exposed to physical, psychological, and sexual violence than other young people of the same age (Svedin et al. 2022). Violence prevention for all is a matter of universal concern. Regarding the extent of violence, previous research shows large variations between studies and between countries (The Swedish National Council for Crime Prevention 2021). Global estimates indicate that about 30% of women have been subjected to either physical and/or sexual intimate partner violence or non‐partner sexual violence in their lifetime (World Health Organization 2024).

### 2.2 The Role of School Health Care

School health services and SHC are two overlapping concepts with different features (WHO and UNESCO 2021). While school health services encompass a wide range of health promotion activities, SHC focuses on the medical and health care services provided within a school environment. School health services are well placed to contribute to adolescent health and development (Baltag et al. 2015). Although many countries have school health services, there is no consensus on what these should include. The absence of guidance on age‐appropriate screening, effectiveness data from robust trials, and cost–benefit analysis of school‐based screening programmes makes decisions regarding how best to allocate SHC personnel time arbitrary (Baltag et al. 2015). Standards for school health services can thus reduce variability and enhance equitable access to health care for young people. Cultural differences, varying legislation, and the fact that not all countries have a well‐developed SHC (WHO and UNESCO 2021) are further challenges influencing what can be achieved regarding sexual health promotion in SHC settings. Globally, girls and young women face barriers, e.g., lack of information, education and consequently knowledge, concerning their sexual and reproductive health, and they therefore avoid seeking health care (Mattebo et al. 2019). Boys and young men also encounter barriers related to their sexual and reproductive health, such as reluctance to admit ill health, restricted access to health facilities, and negative stereotypes of male clients among providers (Aantjes and Govender 2024). Many transgender and non‐binary youths experience stigma and discrimination in accessing health care and find sex education at school heteronormative and not applicable to them (Chong et al. 2021).

Swedish studies have found that boys and young men have less knowledge of, and to a lesser extent use, sexual and reproductive health services and account for only 10%–15% of the patient visits at the youth clinics (Grandahl et al. 2019; Pettersson and Baroudi 2024; Swedish Association of Local Authorities and Regions 2018). In addition to youth clinics, schools have an important role in offering sexual health promotion services and thus reach young people of all genders. The latest report on sexual knowledge, attitudes and behaviour of young people (aged 16 to 29 years) in Sweden highlights the role of SHC and stresses its potential in the efforts to reach equitable SRHR among young people (The Public Health Agency of Sweden 2025). The report showed that 60% of youths had at least one health dialogue with the school health service during their school years, and that 34% of these pupils had been asked questions about sexuality and relationships during the health dialogue. The study indicates that SHC is an untapped resource for promoting young people's SRHR, as 40% had not had such a dialogue.

Educational and health sectors, including school‐based and school‐linked health and social services, can work together to reach large numbers of young people with comprehensive sexuality education (Plesons et al. 2019). Factors that hinder the implementation and integration of more structured SRHR work within Swedish SHC are a lack of leadership engagement (i.e., a lack of support from headmasters); a lack of resources (i.e., structural prerequisites are in place, but SRHR‐specific templates are lacking); a lack of support from teachers and SHC colleagues (i.e., a lack of collaboration and unclear roles); and a lack of research‐informed policies that underline practice (Arvidsson et al. 2024; Bramhagen and Lundström 2022). These barriers are similar to obstacles found in general (not SHC specific) sexual and reproductive health work on a global level, barriers attributed to financial, staffing and time constraints and policy and health systems barriers (Gruskin et al. 2021). To overcome these barriers, several issues need to be addressed. One way to support sexual health promotion for youths could be by working systematically with evidence‐based methods in SHC (WHO and UNESCO 2021).

SEXIT is an evidence‐based method for systematic use that has been partially implemented in SHC but not formally evaluated in this setting. Implementation research has found that whether professionals take up and are able to sustain an improvement or change depends on how well the change fits the adopting people, the organisation, and its context (Miller et al. 2021). Implementation is also affected by several success factors, such as training, mentoring, and clear leadership, but also barriers, such as lack of resources, lack of clarity in the work, and lack of loyalty to the suggested change (Fixsen et al. 2005; Roland 2017). Additionally, the change might be welcomed by personnel but be incompatible with the organisation's systems or policies (Ovretveit et al. 2017). In the context of health care interventions, the terms ‘acceptability’, ‘appropriateness’ and ‘feasibility’ can be used when evaluating the outcomes of new methods and services (Proctor et al. 2023). Acceptability refers to whether an intervention is viewed favourably by its intended users; appropriateness pertains to whether an intervention is suitable within a particular context; and feasibility examines how feasible it is to implement an intervention within a given setting (Proctor et al. 2023). As implementation is complex, it is relevant to explore implementation patterns when methods are being introduced in new contexts (Ovretveit et al. 2017). In this study, the focus is mostly on appropriateness and feasibility, with an assumption that professionals experiencing a new method as valuable and feasible in the setting will be more likely to use it regularly.

### 2.3 SEXIT as a Method to Improve SRHR Among Young People

SEXIT is a systematic method for identifying young people exposed to, or at risk of, sexual ill health and violence, and to offer them adequate support. SEXIT was first pilot‐tested at youth clinics in Sweden (outpatient clinics for young people 13–25 years of age) in 2016 (Hammarström 2021). Youth clinics and SHC are the main settings in which SEXIT is used, but the method is also used at state care institutions for young people with severe psychosocial problems (e.g., substance use, criminality), in care for young people with substance use disorders, and in specialist psychiatric care for children and young people (The Gothenburg Region 2020).

SEXIT has been validated through interview studies with young people visiting youth clinics and professionals working there, and has been found useful and feasible in this setting (Hammarström et al. 2019, 2021, 2022). Youth clinic professionals (mostly midwives, but also counsellors and psychologists) have reported that their systematic use of SEXIT ensures consistency and quality in their assessments and improves their identification of at‐risk young people (Hammarström et al. 2021). The SEXIT questionnaire and the follow‐up conversation are perceived as effective tools for addressing sexual health, sexual ill health and risk‐taking by young people (Hammarström et al. 2022).

The statutory school health dialogue is a pupil‐centred dialogue to gain knowledge about and insight into health and lifestyle (The National Board of Health and Welfare 2023a). Within the SHC setting, SEXIT training has been offered to school nurses in Sweden for use in their health dialogues with pupils in grades 8 and 11 (ages 13 and 16), first in a small pilot study in 2017 (The Gothenburg Region 2020), and from 2023 more regularly in most parts of the country (Knowledge Centre for Sexual Health 2023). Qualitative studies suggest that SEXIT has helped school nurses identify risks for sexual ill health at an early stage and school nurses expressed the view that SEXIT resulted in more meaningful conversations with young people about sexuality, risk‐taking and experience of violence (Wilhsson et al. 2023). SEXIT also helped SHC professionals to act as educators promoting sexual health and freedom from violence, but a lack of supportive leadership or a unified approach hindered effective implementation (Håkansson et al. 2024). A more unified approach can be found in one region in Sweden, where youth clinics, child and adolescent psychiatric services, and SHC have been working with SEXIT in all municipalities since 2021 (Region Jämtland Härjedalen 2024).

There are few structured sexual health screening tools assessing sexual risk, and they often have a limited focus on STI testing only, such as SRBS (Sexual Risk Behaviours Scale) (Fino et al. 2021), or assess a variety of health factors, of which sexuality is only one, such as HEEADSSS (Home, Education/Employment, Eating, Activities, Drugs, Sexuality, Safety and Suicide/Depression) (Rizk et al. 2023). To the best of our knowledge, there is no method comparable to SEXIT, where the aim is to promote sexual health and detect experiences of sexual ill health and violence.

Overall, previous research indicates that SEXIT can be systematically used in the school nurses' health dialogues, providing a foundation for identifying adolescents at risk of sexual ill health. However, further research is required to enhance understanding of the implementation of SEXIT, including potential barriers and its perceived value within SHC.

## 3 The Study

### 3.1 Aim

The aim of the study was to explore implementation patterns and the perceived value of the SEXIT method in SHC settings in Sweden.

## 4 Methods

### 4.1 Design, Recruitment and Data Collection

To reach a comprehensive understanding, the study adopted a mixed method design using an online questionnaire with closed and free‐text response options, which were analysed in an integrated manner (Doyle et al. 2009). The Checklist for Reporting Of Survey Studies (CROSS) (Sharma et al. 2021) has been followed (Data S1). Since 2021, approximately 500 SHC professionals (mainly school nurses but also school counsellors) from lower secondary and upper secondary schools across Sweden have been trained in using SEXIT. In conjunction with this training, these professionals provided an e‐mail address and gave their consent to be contacted by the Knowledge Centre for Sexual Health in the region of Västra Götaland, Sweden, the organisation that provided the training. The total number of e‐mail addresses was 478, but 42 e‐mails bounced back due to old or invalid e‐mail addresses. All those who received this training and had an active e‐mail address (*n* = 436) were invited via e‐mail to participate. The questionnaire was sent out via e‐mail on two occasions (in March and May 2024), with two follow‐up reminders sent at 10‐day intervals after each occasion.

### 4.2 Questionnaire

The questionnaire was designed to evaluate the use and perceived value of SEXIT in the SHC setting. After the questionnaire was designed, face validity was assessed by a group of five SHC professionals with extensive experience in working with the SEXIT method. This led to minor language revisions and the deletion of some questions to better focus on the purpose of the study.

The questionnaire had three parts with a total of 72 questions. The first part (items 1–13) concerned profession, age, working conditions (e.g., number of pupils the professionals were responsible for in their schools), and general questions about the use of SEXIT (e.g., asking what year they attended the SEXIT training). Part two (items 14–40) contained questions taken from the Professionals' Attitudes towards addressing Sexual Health Extended questionnaire, PA‐SH‐Ext (Areskoug Josefsson 2022). The PA‐SH‐Ext has been psychometrically validated and used in several settings to assess attitudes towards addressing sexual health as a professional (Elnegaard et al. 2022; Frawley et al. 2022). The third part (items 41–72) focused on SHC professionals' experience of working with SEXIT. Most questions were answered on a five‐point Likert scale (disagree/partly disagree/partly agree/agree/strongly agree). In the first and third parts of the questionnaire, there were opportunities to provide free‐text answers to some of the questions, and these were used to obtain more in‐depth knowledge. All respondents in the study had attended the SEXIT training, but only those who had also used SEXIT in their work answered part three. In this paper, only the responses from parts one and three (sequential in the questionnaire) are analysed as they correspond to the study aim; part two will be analysed in another study. The questionnaire was created using esMaker, and the average response time was 12 min.

### 4.3 Data Analysis

The analysis was based on a sequential mixed method design (Ivankova et al. 2006), which means that we started with a quantitative analysis followed by a qualitative analysis and then the results were integrated. All quantitative data were transferred to SPSS and analysed with descriptive statistics such as frequencies and percentages. The items, which had responses on a scale of five, were trichotomized. The response options ‘disagree’/‘partly disagree’ (negative answers) were merged and the response options ‘agree’/‘strongly agree’ (positive answers) were merged. The response option ‘partly agree’ remained a separate group. Trichotomization was chosen to improve interpretability (DeCoster et al. 2009).

The analysis of the free‐text answers was performed using deductive content analysis (Krippendorff 2013), and examples that provided answers relevant to the aim (implementation patterns and perceived value) were sought. The questionnaire had four different free‐text response items: (1) If you do not use SEXIT, comment on why; (2) If you do not use SEXIT with some pupils, why and with which group(s)?; (3) If you skip questions in SEXIT, which question(s)?; and (4) Other comments. One person in the research team coded the free‐text responses in the questionnaire and created initial categories (e.g., time concerns, reasons for not using SEXIT, reasons for starting to use SEXIT later, using similar questions instead of SEXIT, SEXIT not suitable, SEXIT an important tool), and a team colleague validated these categories. Consensus regarding where these categories and related quotes fitted the results presentation was reached through discussions in the whole team.

### 4.4 Ethical Considerations

The study was approved by the Swedish Ethical Review Authority (2023‐06691‐01). E‐mail addresses for participants (those who had completed the SEXIT training programme) were obtained from a list stored by Region Västra Götaland. Region Västra Götaland processes all personal data in accordance with current Swedish data protection legislation. Participants received study information via e‐mail, in which they were informed that participation was voluntary, that they could choose not to answer the questionnaire, and that the data would be handled in accordance with procedures established by the Region of Västra Götaland; it would be presented on an aggregated level, and participant anonymity would be ensured. The information also included written information with contact details for the research coordinator who could answer any questions.

## 5 Results

In total, 115 professionals trained in using SEXIT answered the questionnaire, see Table 1, providing a response rate of 26%, based on the 436 invitations sent. Of these, 61% (*n* = 70) used the method and responses from this group are the main data source. Of the 70 respondents who said that they used SEXIT, 60 answered the items in Part 3 (items 41–72) concerning how they used SEXIT. Therefore, the part of the results focusing on the perceived value of SEXIT is based mainly on the 60 respondents who had both taken the training and used SEXIT. Data from non‐users are presented when relevant. Throughout, quotes from the free‐text responses are used to illustrate the quantitative findings (Table 2).

**TABLE 1:** Characteristics of the participants trained in using SEXIT and distribution of users/non‐users (*n* = 115).

Variable	Number
**Gender**	
Female	112
Male	3
Other	0
Age	Range 27 to 69 years. Mean age 49.5
**Profession**	
School nurse	78
School counsellor	34
School physician	1
Specialised educator	1
Head of student health services	1
**School grade** ^a^	
Secondary school	62
Upper secondary school	51
Special school^b^	8
**Use of the SEXIT method**	
User	70
Non‐user	45

^a^ Multiple answers possible.

^b^ Pupils with intellectual disabilities.

**TABLE 2:** Questionnaire responses from SHC professionals who attended the SEXIT training and worked with the method.

	Disagree/partly disagree	Partly agree	Agree/strongly agree
	% (*n*)	% (*n*)	% (*n*)
41 Before the interviews I inform the pupils that SEXIT will be used	7 (4)	10 (6)	82 (49)
42 I always go through the answers in SEXIT with the pupil	2 (1)	10 (6)	87 (52)
43 I ask follow‐up questions when the pupil has answered something that, according to the SEXIT method, signals risk exposure	0 (0)	0 (0)	97 (58)
44 I have used the SEXIT visual and linguistic support in easy Swedish and/or in other languages	77 (46)	10 (6)	12 (7)
45 Pupils choose to answer the SEXIT questions	2 (1)	10 (6)	87 (52)
46 It feels easy to use SEXIT	0 (0)	10 (6)	88 (53)
47 Using SEXIT involves addressing sensitive topics	75 (45)	18 (11)	5 (3)
48 Using SEXIT in the conversations gives me a better picture of the pupil's situation	2 (1)	5 (3)	92 (55)
49 SEXIT helps me identify pupils who have experience of sex for money	15 (9)	15 (9)	65 (39)
50 SEXIT helps me identify pupils who have experienced sexual ill health	7 (4)	12 (7)	78 (47)
51 SEXIT helps me identify pupils who have experienced violence	0 (0)	13 (8)	83 (50)
52 SEXIT has given me a better understanding of how sexual ill health and violence can affect pupils	5 (3)	25 (15)	67 (40)
53 Before I started using SEXIT, I asked all pupils questions about sexual risk‐taking	58 (35)	27 (16)	13 (8)
54 Before I started using SEXIT, I asked all pupils about their experience of violence	38 (23)	33 (20)	27 (16)
55 I have previously lacked a questionnaire to help me ask questions about sexual health and risk exposure	7 (4)	20 (12)	72 (43)
56 SEXIT helps me ask questions that otherwise feel difficult to raise	3 (2)	10 (6)	85 (51)
57 SEXIT is a suitable method to use in school health care	0 (0)	3 (2)	97 (58)
58 I feel confident in using SEXIT	0 (0)	8 (5)	92 (55)
59 I sometimes skip SEXIT with some pupils	63 (38)	15 (9)	18 (11)
61 I sometimes skip one or more questions in SEXIT	92 (55)	2 (1)	5 (3)
63 I am supported by the principal and/or school health manager in working with SEXIT	12 (7)	15 (9)	70 (42)
64 I am supported by colleagues (in the SHC) in working with SEXIT	12 (7)	18 (11)	67 (40)
65 We collaborate in the school health team around what emerges through SEXIT in detecting sexual health and violence	35 (21)	22 (13)	37 (22)
66 I know what action to take when sexual ill health is recognised	2 (1)	10 (6)	88 (53)
67 I know what action to take when violence is recognised	2 (1)	5 (3)	93 (56)
68 The use of SEXIT has been questioned by parents	95 (57)	2 (1)	0 (0)
69 The use of SEXIT has been questioned by pupils	90 (54)	8 (5)	0 (0)
70 The SEXIT manual supports the work	3 (2)	8 (5)	88 (53)
71 I would recommend SEXIT to other schools	0 (0)	3 (2)	97 (58)

*Note:* The total sample of respondents was 60, and for each item, the exact number of respondents has been presented to clarify the number of non‐respondents per item. Percentages are rounded to whole numbers. Questions 60, 62 and 72 had free‐text response options. No responses were excluded.

### 5.1 Patterns of Implementation of SEXIT

Among SHC professionals trained in using the method, 63% used SEXIT during their regular health dialogues. Of those who did not use SEXIT, 56% planned to do so. The most common reasons for non‐use were that SEXIT was already used by another colleague in health dialogues, i.e., counsellors responding that the nurse used SEXIT (18%), and not feeling adequately prepared (16%). In the free‐text responses, concerns about not being prepared were expressed:

I was unsure how to get started. I see the point and would like to, but I did not start right away, and I have difficulty getting the time required. I have very little time to work on prevention and health promotion. (School nurse, not using SEXIT)

The majority of the professionals using SEXIT reported that they followed the method as intended, i.e., they informed the pupils before the health dialogue that SEXIT would be used (82%) and discussed the answers with the pupils (87%). All respondents asked follow‐up questions when the pupil gave an answer that, according to the SEXIT method manual, indicated risk exposure, and 92% did not skip any of the questions in the SEXIT questionnaire.

When asked if they had used the visual and linguistic support in easy‐to‐read Swedish and/or in other languages, 12% said that they had. There was criticism that the language used in SEXIT was too difficult:

The questions asked in the questionnaire would need to be worded a little more simply and a little clearer to ensure that all pupils understand the questions correctly. (School nurse, using SEXIT)

Almost one‐fifth (18%) of those who used the questionnaire did not use it with all pupils. In the free‐text section, reasons given were that pupils declined to answer it, that the participant judged the pupil not to be at risk, or that SEXIT was not suitable because it was similar to other questionnaires SHC professionals were already using, and they had to prioritise which questionnaires to use. Some free‐text responses related to using SEXIT with all youths revealed uncertainty among professionals, for example about their own capacity to help the pupil, as well as uncertainty about whether SEXIT was appropriate for pupils who they suspected had been sexually abused and were already receiving support elsewhere:

Pupils where I have the understanding that they have been sexually abused and already have mental health support/treatment … I am considering offering them the SEXIT questionnaire anyway, letting them choose just like any other pupil, but am unsure if it benefits them or not. I am not sure if I am able to deal with any more severe reactions. (School counsellor, using SEXIT)

There were also other reasons for not using SEXIT. Excluding pupils in state care was highlighted by one of the respondents:

At the time, I was working in a secondary school and wanted to use SEXIT. The principal said no. Now I work in a small resource school where SEXIT is not suitable. Most of the pupils are placed in institutional care. (School nurse, not using SEXIT)

Just over one quarter (27%) of the professionals said they had asked all pupils about their experience of violence also before implementing SEXIT. Few (13%) had asked about sexual risk‐taking before the implementation. Almost three‐quarters (72%) stated that they had previously lacked a questionnaire to use when addressing sexual health and risk exposure.

When asked if they received support from the principal and/or head of the SHC team in working with SEXIT, 70% said that they did, and 67% reported that they received support from other colleagues in the team. The proportion of respondents collaborating within the SHC team (37%) regarding SEXIT findings was similar to the proportion of respondents not doing so (35%). A high proportion of the professionals (88%) said they knew what action to take when sexual ill health issues were identified, and that they knew what action to take when violence was identified (93%).

To summarise, patterns of implementation show that the majority of SHC professionals were using SEXIT as intended, and follow‐up questions were asked when the pupil's answers indicated risk. However, one‐fifth did not use it with all pupils, which indicates that SEXIT is not always used as intended.

### 5.2 Perceived Value of SEXIT

In the group using SEXIT, 78% of the professionals responded that the method helped them identify pupils with sexual ill health, while 83% said it helped identify pupils with experience of violence. Most (92%) thought that SEXIT helped them gain a better understanding of the pupils' situation.

Most of the professionals (88%) found SEXIT easy to use and 92% felt confident in using it. Only 5% of the professionals stated that using SEXIT involved addressing sensitive topics. When asked if pupils chose to answer the SEXIT questionnaire when offered it, 87% said that pupils did. The questionnaire seemed widely accepted as only very few reported being questioned by parents and pupils regarding SEXIT.

Over half (67%) of the professionals who used SEXIT responded that it had given them a better understanding of how sexual ill health and violence affect young people. Almost all (97%) considered SEXIT to be an appropriate method in SHC work pertaining to these issues. The majority (85%) also considered that SEXIT helped them to ask questions that otherwise they felt were difficult to raise. Almost all (97%) said they would recommend SEXIT to colleagues at other schools, and in the free‐text responses the following explanation for this was given:

I have worked in school health care before SEXIT was introduced in the municipality, but I think it's a very simple and helpful tool to easily raise issues that pupils may find difficult/sensitive. In addition, you get a kind of overall picture of how the pupils are doing and their knowledge about sexual health. (School nurse, using SEXIT)

One issue raised in the free‐text responses was the amount of work and time required for using SEXIT. Of those who did not use SEXIT (*n* = 45), 13% answered that the reason was that the method was too time‐consuming, as illustrated in the following quote:

Today's health dialogue takes about an hour in conversation. There is no room to add more time if I am to fit in all the health dialogues. However, we have added some aspects from the SEXIT questionnaire to our own health dialogue instrument. (School nurse, not using SEXIT)

In the free‐text responses, also opposite views were shared, where lack of time was not regarded as a problem:

I use SEXIT in health dialogues in year 8 and it takes very little time for most pupils. Those for whom it does take time gain so much in the end, so it's worth it! We document SEXIT as ‘SEXIT dialogues’ so it takes no time at all, and no sensitive information is recorded. Subsequent work is of course documented in the records in the usual way. (School nurse, using SEXIT)

In the free‐text responses, several professionals said that they did not use SEXIT but used similar questions in their health dialogues instead, indicating that they saw no need for SEXIT. Experiences that SEXIT complements existing health questionnaires and provides a more in‐depth understanding were also highlighted:

I am glad that I tested it on some pupils. I was quite negative before because I felt that there were already many questions in the health questionnaire but felt that the SEXIT questionnaire provided much more in‐depth information and none of the pupils found it difficult. (School nurse, using SEXIT)

The importance of using SEXIT systematically with all pupils was also mentioned:

By using it in connection with the health dialogues and preparing the pupils before the dialogues, it becomes natural, and by reaching out to everyone, you also find out more about those who you may not have suspected were vulnerable. (School nurse, using SEXIT)

However, in the free‐text responses, some reported not seeing the value of using SEXIT if they judged the pupil not to be at risk. In response to the question regarding those with whom SEXIT was not used, the following answer was provided:

Pupils seeking help for problems that cannot be clearly linked to SEXIT‐related symptoms. (School counsellor, using SEXIT)

The connection between health and school performance was stressed in the following free‐text response:

A useful material that shows how important it is to have a common thread in education from children to young adults. I have used the results from the questionnaires and presented them to the SHC team and teachers, thereby also highlighting the important work that takes place in education about pupils' health. By working on this, talking more openly and creating a climate in which, for example, violence is recognised, both by those who perpetrate it and those who experience it, we can create change, for some in any case. (School nurse, using SEXIT)

The perceived value of SEXIT reported was that SHC professionals found it to be an appropriate and useful tool that helped them gain a better understanding of the pupil's situation. Time was an issue raised in the free‐text responses where some expressed that SEXIT took too much time, although others expressed that using the method was not time‐consuming. Some professionals mentioned the importance of using SEXIT with all youths, while others used it selectively.

## 6 Discussion

### 6.1 Patterns of Implementation of the SEXIT Method in the SHC

The use of SEXIT found among SHC professionals is in line with the guidelines in the SEXIT manual, such as informing pupils that SEXIT will be offered and going through the answers with the pupil (Hammarström et al. 2021). Although the results of the study illustrate that SHC professionals are in favour of using SEXIT, we also found they sometimes opted out of using the method. One reason given was that the same or similar questions were asked when using other general health questionnaires. In Sweden, however, there is no common model for what questions should be included in a health dialogue (The National Board of Health and Welfare 2023a); therefore, this needs further exploration. As topics raised in the health dialogue can vary between schools, they can include questions about sexual health and/or experience of violence, but this is not mandatory (The Public Health Agency of Sweden 2023). Borrowing questions from SEXIT and only using parts of it compromises the consistency and quality gained from its systematic use (Hammarström et al. 2021). Another reason given for not using SEXIT was that there was no time to do so within existing health dialogues. This echoes research that has shown that the lack of time allotted for the statutory health dialogues is perceived as a dilemma where school health nurses want the conversations to focus on the pupil's questions and needs, and fear that using health questionnaires takes time from the pupils' own agendas (Kostenius 2021). However, some respondents in our study reported that SEXIT saved time, as has previously been described elsewhere (Hammarström et al. 2021; Håkansson et al. 2024).

The participants considered SEXIT easy to use in the SHC setting, which is in agreement with professionals' perceptions of SEXIT in Swedish youth clinics (Hammarström et al. 2021). However, the free‐text responses in the present study illustrate that SHC professionals sometimes did not use SEXIT with some pupils. One reason may be that professionals feel that the pupil is not ready to talk about the topic; another is that professionals do not know how to handle answers they receive. This insecurity is also found in a study where school nurses described ambivalence in asking questions about child sexual abuse, and by omitting questions about child sexual abuse, they avoided professional insecurity and the emotional consequences of the pupil's answers (Engh Kraft et al. 2017). It is important that the professionals and pupils collaboratively explore the nature of violence (Cater et al. 2014; Havard et al. 2023). Often there are multiple violent situations occurring simultaneously, and the pupil might have had several previous experiences. The SEXIT manual provides general recommendations regarding violence, but each school must ensure that there are proper procedures in place, such as referral mechanisms, although we have not studied this area.

As there is a risk of secondary traumatic reactions among professionals who work with sexually violated clients (von Mentzer et al. 2024), this is relevant to address when implementing SEXIT in the future. To support the implementation of SEXIT after the one‐day training, SHC staff can also attend digital booster sessions organized three to four times a year where participants share experiences with each other about working with SEXIT. Sometimes these sessions also include a short lecture on a current theme. Each session is thus unique, and there is no limit to the number of times one can attend. The number of participants in these post‐training sessions has been significantly lower than those who attended the one‐day training (Region Västra Götaland 2025). These booster sessions have not been explored in research, and their significance as post‐training support remains unclear.

The number of SHC personnel using the SEXIT visual and linguistic support in easy Swedish and/or in other languages was low. This support is suitable for pupils with an immigrant background and/or intellectual difficulties. Statistics show that 27% of pupils in compulsory education in Sweden during the academic year 2023–2024 had a foreign background (The National Agency for Education 2024). Having a foreign background does not per se indicate a language barrier, but previous research shows that young people in Sweden with migration experience report lower access to sexual and reproductive health services (Amroussia et al. 2024; Baroudi et al. 2022). Our findings are similar to another study where SHC professionals did not use SEXIT with pupils where there were language barriers, as they were concerned that language and cultural barriers could lead to misinterpretation of issues with young people of immigrant background, and they avoided using SEXIT with pupils who had intellectual disabilities, assuming that the disability would hinder their full comprehension of the questions (Håkansson et al. 2024). Pupils with intellectual disability in special schools can be vulnerable to sexual ill health, and they often receive inadequate sexuality education (Daigneault et al. 2023; Lafferty et al. 2012; Löfgren‐Mårtenson and Ouis 2019; Paulauskaite et al. 2022). In line with our findings, previous research shows that most professionals avoided using SEXIT with pupils who had intellectual disabilities, assuming that the disability would hinder their full comprehension of the questions (Håkansson et al. 2024).

The results also show that some SHC professionals only use parts of the SEXIT questionnaire. Avoiding the use of SEXIT if the pupil already receives support, e.g., from mental health care services, is problematic as the professional cannot know what has been discussed with another health care provider. Also, mental health providers may be hesitant to address sexual health issues with young people (Bungener et al. 2022), and young people found it reassuring to know that SEXIT was used with all youths (Hammarström et al. 2022). The findings suggest that SHC professionals consider SEXIT unsuitable for pupils in institutions (such as special residential homes). This is another example of professionals excluding some pupils, which is a serious concern as young people in institutional care have sexual health care needs to a higher extent and simultaneously limited access to sexual and reproductive health care compared to same‐aged peers (Schindele and Lindroth 2021). Excluding pupils due to their gender identity does not appear in the findings.

The patterns of implementation point out that care provided by the SHC team does not reach all youths; socially marginalised and vulnerable youths may not get the support they need. These exclusionary practices warrant further investigation. In future SEXIT training and booster sessions, it is essential to focus more on how to ensure implementation patterns that support professionals' use of the method for all pupils, especially structurally marginalised and vulnerable youths, to ensure equitable SHC dialogues. There is a need for future research on SEXIT and its implementation with young people with intellectual disabilities, as well as those with migrant backgrounds, mental health problems and in state care. SHC professionals cannot see who is at risk; thus, they must address sexual health and experiences of violence with all youths to ensure equitable care.

### 6.2 The Perceived Value of the SEXIT Method in SHC

The responses show that the SHC professionals felt confident in using SEXIT and that most of those who had not yet started using the method planned to do so. Using SEXIT in the health dialogues was found to help SHC professionals get a better understanding of the pupil's situation, which is in line with previous research (Håkansson et al. 2024). Our findings indicate that SEXIT complemented the regular health assessments, which often lack specific inquiries related to sexual health and experiences of violence. This echoes earlier results that SEXIT can improve consistency and quality of health dialogues (Håkansson et al. 2024; Kostenius 2021).

Most professionals who used SEXIT felt that they had support from both colleagues and the principal in working with the method. At the same time, one‐third said that they did not cooperate within the SHC when information about sexual health and experiences of violence emerged in the conversations. Similar results exist, where SHC professionals, SHC team managers and headmasters considered SRHR as an urgent topic but said that collaboration on SRHR‐related work was rare, both within the SHC team and between the SHC team and teachers (Arvidsson et al. 2024; Bramhagen and Lundström 2022). This paradox needs further explorations, especially since support from colleagues is related to capabilities for handling clinical problems (Sodeify and Habibpour 2021). However, school nurses and school counsellors in Sweden are partly governed by different legal provisions—a situation that problematises information sharing and adds to the complexity of a multidisciplinary and collaborative approach when addressing sexual and reproductive health in school settings (The National Board of Health and Welfare 2023b). This is unfortunate since addressing sexual and reproductive health in school settings requires a multidisciplinary and collaborative approach. There is a substantial body of research on the vital role of leaders in the implementation of health promotion efforts in schools (Adams et al. 2023; Leksy et al. 2023). Learning and implementing a new method are facilitated by professionals working together, where the role of the leader is to develop a collaborative culture (Billett 2002). Our findings are in line with this, and the role of the school or SHC team leader needs to be further explored in future implementation and research related to the use of SEXIT.

## 7 Limitations

A limitation of this study is the relatively low response rate, which influences the generalisability of the findings. One possible explanation for the low response rate could be high workload in the group, or that those who did not use SEXIT (despite being trained) chose not to respond. A larger number of respondents would have strengthened the results, but at the same time, a response rate of 26% is what one can expect in this kind of survey (Kidd et al. 2021). Interest in a particular topic can affect the outcome of a survey (Groves et al. 2004). The overall positive outcome of the study may be related to the fact that the participants had an interest in sexual and reproductive health and rights as well as favourable attitudes towards the SEXIT method. As the responses confirm previous research in the field of SHC and sexual health promotion, the findings can be of value for clinical practice and constitute a basis for future research and implementation in contexts with similar views and legislation on sexual and reproductive health and rights for all.

## 8 Conclusions

The main findings include that most SHC professionals who had completed SEXIT training also use the method in their work and perceive the method to facilitate discussions about sexual ill health and experiences of violence. The approach thus contributes to structured work and facilitates dialogue on sexual ill health and violence, indicating the usefulness of the SEXIT method in SHC.

Although most pupils were offered SEXIT in health dialogues, the method's visual and linguistic support was seldom used, indicating that SHC professionals avoid using SEXIT with structurally marginalised and vulnerable youths. These findings indicate a lack of equity in sexual health promotion, which is a topic for future research. Relatedly, there is a need to further explore which types of support can enable SHC professionals to use SEXIT with all pupils.

### 8.1 Relevance to Clinical Practice

The results may be of use to SHC professionals and SHC leaders, as they can serve as a basis for understanding the value of using a structured tool for sexual health promotion through communication about sexual ill health and violence with youths. The paper points out the strengths of using a systematic method, but also the pitfalls that professionals may encounter, such as failing to reach out to all youths. The results also show that implementing new methods takes time, and that preparedness is required (among SHC professionals, their colleagues, and management), not only for the implementation process but also for SHC professionals' reactions when pupils provide information about sexual ill health, abuse, and violence.

## Author Contributions

Conceptualization: Per Andreas Persson, Kristina Areskoug Josefsson, Malin Lindroth, Sofia Hammarström. Data Curation: Per Andreas Persson, Sofia Hammarström. Formal Analysis: Per Andreas Persson, Kristina Areskoug Josefsson. Funding Acquisition: Per Andreas Persson, Kristina Areskoug Josefsson, Malin Lindroth, Sofia Hammarström. Investigation: Per Andreas Persson. Methodology: Per Andreas Persson, Kristina Areskoug Josefsson, Malin Lindroth, Sofia Hammarström. Project Administration: Per Andreas Persson. Resources: Not applicable. Software: Not applicable. Supervision: Kristina Areskoug Josefsson, Malin Lindroth, Sofia Hammarström. Validation: Per Andreas Persson, Kristina Areskoug Josefsson, Malin Lindroth, Sofia Hammarström. Visualisation: Per Andreas Persson. Writing – Original Draft Preparation: Per Andreas Persson. Writing – Review and Editing: Per Andreas Persson, Kristina Areskoug Josefsson, Malin Lindroth, Sofia Hammarström.

## Conflicts of Interest

The authors declare no conflicts of interest.

## Supporting information


**Data S1:** jocn70135‐sup‐0001‐Supinfo01.docx.

## Data Availability

The data supporting the findings of this study are available from the corresponding author upon reasonable request.
