# Supplementary material for: Implementation Patterns and Perceived Value of the SEXIT Method in School Health Care
Source: J Clin Nurs. 2025 Oct 13;35(4):1556–68. doi: 10.1111/jocn.70135 (PMC12964520; doi:10.1111/jocn.70135)
Supplement: Supplementary file 1 — Data S1: jocn70135‐sup‐0001‐Supinfo01.docx. [file JOCN-35-1556-s001.docx]

**Supplement 1. Information about experts included in the Delphi process**

| **Name** | **Affiliation** | **Field of expertise** | **Years of experience** | **Number of surveys involved*** |
| --- | --- | --- | --- | --- |
| Christine L. Paul | University of Newcastle Faculty of Medicine and Health Sciences, Priority Research Centre for Health Behaviour, Callaghan, Australia | Behavioural science | 20 | 40 |
| Janette Kwok | Queen Mary Hospital Hong Kong, Department of Pathology, Hong Kong, China | Transplantation, population genetics, | 25 | 10 |
| Juntra Karbwang | Nagasaki University, Department of Clinical Product Development, Nagasaki, Japan | Clinical Pharmacology | 50 | Not reported |
| Chiara de Waure | Universita degli Studi di Perugia, Perugia, Italy | Epidemiology, Public Health, Health Technology Assessment | 11 | 18 |
| Frances J. Drummond | University College Cork, Cancer Research @ UCC, Cork, Ireland | Cancer research | 15 | 14 |
| Masahiro Hashizume | Nagasaki University, Institute of Tropical Medicine, Nagasaki, Japan | Environmental Epidemiology | 20 | Not reported |
| Yoshiyuki Kizawa | Kobe University School of Medicine, Kobe, Japan | Palliative Medicine | 29 | 40 |
| Erik Taal | University of Twente, Department of Psychology, Health and Technology, Enschede, Netherlands | Health psychology | 37 | 25 |
| Joeri Vermeulen | Vrije Universiteit Brussel (VUB), Brussels, Belgium and Erasmus Brussels University of Applied Sciences and Arts, Brussels, Belgium | Public Health & Midwifery | 20 | 10 |
| Gillian H.M. Lee | Faculty of Dentistry, The University of Hong Kong, Pokfulam, Hong Kong | Paediatric Dentistry | 15 | 10 |
| Adam Gyedu | Kwame Nkrumah University of Science and Technology, Department of Surgery, Kumasi, Ghana | Surgery | 13 | 10 |
| Kien Gia To | University of Medicine and Pharmacy at Ho Chi Minh City, Faculty of Public Health, Ho Chi Minh City, Viet Nam | Public Health | 17 | 40 |
| Martin L. Verra | UniversitatsSpital Bern, Department of Physiotherapy, Bern, Switzerland | Physiotherapy | 34 | Not reported |
| Évelyne M. Jacqz- Aigrain | Hopital Robert-Debre AP-HP, Department of Pediatric Pharmacology and Pharmacogenetics, Paris, France | Paediatric Pharmacology | 30 | 8 |
| Wouter KG Leclercq | Máxima Medical Center, Veldhoven, Department of Surgery, Veldhoven, Netherlands | General surgery | 16 | 20 |
| Simo T. Salminen | Tyoterveyslaitos, Helsinki, Finland | Occupational safety | 32 | 15 |
| Cathy Donald Sherbourne | RAND, Santa Monica, USA | Health Services Research | 43 | 37 |
| Barbara Mintzes | The University of Sydney, Charles Perkins Centre, Sydney, Australia | Pharmaceutical policy | 20 | 5 |
| Sergi Lozano | The Catalan Institute of Human Paleoecology and Social Evolution (IPHES), Tarragona, Spain | World Economy | 20 | Not reported |
| Ulrich S. Tran | University of Vienna, School of Psychology, Department of Cognition, Emotion, and Methods in Psychology, Vienna, Austria | Methods in Psychology | 15 | 50 |
| Ana Marušić | University of Split School of Medicine, Croatia | Evidence-based Medicine | 30 | 20 |
| Matsui Mitsuaki | Nagasaki University School of Tropical Medicine and Global Health, Japan | Global Health, Reproductive Health | 30 | 15 |
| Mohammad Karamouzian | School of Population and Public Health, University of British Columbia, Vancouver, BC, Canada | Public Health & Epidemiology | 10 | 20 |
| David Moher | Ottawa Hospital Research Institute, Canada | Clinical Epidemiology | 36 | PRISMA and Consort guidelines |

*****Self-reported by the expert panel members.

**Supplement 2. Sample PubMed search strategy**

1. (("web-based"[Title/Abstract] OR "online")[ Title/Abstract]) AND (("survey*"[ Title/Abstract] OR "questionnaire*")[ Title/Abstract])
2. (survey[Title/Abstract] or questionnaire[Title/Abstract]) AND ("Cancer Journal for Clinicians"[Journal] OR "The Lancet Oncology"[Journal] OR "New England Journal of Medicine"[Journal] OR "The Lancet"[Journal] OR "The Lancet Neurology"[Journal] OR "The Lancet Infectious Diseases"[Journal] OR "Nature Medicine"[Journal] OR "Nano Today"[Journal] OR "Cancer Cell"[Journal] OR "Alzheimer's and Dementia"[Journal] OR "Immunity"[Journal] OR "Journal of the American College of Cardiology"[Journal] OR "Journal of Experimental Medicine"[Journal] OR "Annual Review of Clinical Psychology"[Journal] OR "Journal of Clinical Investigation"[Journal] OR "Genome Research"[Journal] OR "Journal of Clinical Oncology"[Journal] OR "Archives of General Psychiatry"[Journal] OR "Molecular Systems Biology"[Journal] OR "Molecular Psychiatry"[Journal] OR "Circulation"[Journal] OR "Science Translational Medicine"[Journal] OR "European Heart Journal"[Journal] OR "Journal of the National Cancer Institute"[Journal] OR "European Urology"[Journal] OR "American Journal of Psychiatry"[Journal] OR "Gut"[Journal] OR "Journal of the American Medical Association"[Journal] OR "Journal of Allergy and Clinical Immunology"[Journal] OR "Gastroenterology"[Journal] OR "Annals of Neurology"[Journal] OR "American Journal of Human Genetics"[Journal] OR "American Journal of Respiratory and Critical Care Medicine"[Journal] OR "Brain; a journal of neurology"[Journal] OR "Hepatology"[Journal] OR "Circulation Research"[Journal] OR "Journal of the American Society of Nephrology : JASN"[Journal] OR "Molecular Aspects of Medicine"[Journal] OR "Acta Neuropathologica"[Journal] OR "World Psychiatry"[Journal] OR "Diabetes Care"[Journal] OR "Annals of Internal Medicine"[Journal] OR "Journal of Hepatology"[Journal] OR "Journal of Cell Biology"[Journal] OR "Clinical Infectious Diseases"[Journal] OR "Cancer Research"[Journal] OR "EMBO Journal"[Journal] OR "Annals of the Rheumatic Diseases"[Journal] OR "JAMA Internal Medicine"[Journal] OR "Blood"[Journal] OR "British Medical Journal" [Journal])
3. (survey* [Title/Abstract] or questionnaire* [Title/Abstract]) AND (recommendation [Title/Abstract] OR reporting [Title/Abstract] OR quality [Title/Abstract])
4. 1 OR 2 OR 3
5. Limit to Humans and Observational Studies

**Supplement 3: Checklist for Reporting Of Survey Studies (CROSS)**

| **Section/topic** | **Item** | **Item description** | **Reported on page #** |
| --- | --- | --- | --- |
| **Title and abstract** | | |  |
| Title and abstract | 1a | State the word “survey” along with a commonly used term in title or abstract to introduce the study’s design. |  |
|  | 1b | Provide an informative summary in the abstract, covering background, objectives, methods, findings/results, interpretation/discussion, and conclusions. |  |
| **Introduction** | | |  |
| Background | 2 | Provide a background about the rationale of study, what has been previously done, and why this survey is needed. |  |
| Purpose/aim | 3 | Identify specific purposes, aims, goals, or objectives of the study. |  |
| **Methods** | | |  |
| Study design | 4 | Specify the study design in the methods section with a commonly used term (e.g., cross-sectional or longitudinal). |  |
|  | 5a | Describe the questionnaire (e.g., number of sections, number of questions, number and names of instruments used). |  |
| Data collection methods | 5b | Describe all questionnaire instruments that were used in the survey to measure particular concepts. Report target population, reported validity and reliability information, scoring/classification procedure, and reference links (if any). |  |
|  | 5c | Provide information on pretesting of the questionnaire, if performed (in the article or in an online supplement). Report the method of pretesting, number of times questionnaire was pre-tested, number and demographics of participants used for pretesting, and the level of similarity of demographics between pre-testing participants and sample population. |  |
|  | 5d | Questionnaire if possible, should be fully provided (in the article, or as appendices or as an online supplement). |  |
| Sample characteristics | 6a | Describe the study population (i.e., background, locations, eligibility criteria for participant inclusion in survey, exclusion criteria). |  |
|  | 6b | Describe the sampling techniques used (e.g., single stage or multistage sampling, simple random sampling, stratified sampling, cluster sampling, convenience sampling). Specify the locations of sample participants whenever clustered sampling was applied. |  |
|  | 6c | Provide information on sample size, along with details of sample size calculation. |  |
|  | 6d | Describe how representative the sample is of the study population (or target population if possible), particularly for population-based surveys. |  |
| Survey  administration | 7a | Provide information on modes of questionnaire administration, including the type and number of contacts, the location where the survey was conducted (e.g., outpatient room or by use of online tools, such as SurveyMonkey). |  |
|  | 7b | Provide information of survey’s time frame, such as periods of recruitment, exposure, and follow-up days. |  |
|  | 7c | Provide information on the entry process:  –>For non-web-based surveys, provide approaches to minimize human error in data entry.  –>For web-based surveys, provide approaches to prevent “multiple participation” of participants. |  |
| Study preparation | 8 | Describe any preparation process before conducting the survey (e.g., interviewers’ training process, advertising the survey). |  |
| Ethical considerations | 9a | Provide information on ethical approval for the survey if obtained, including informed consent, institutional review board [IRB] approval, Helsinki declaration, and good clinical practice [GCP] declaration (as appropriate). |  |
|  | 9b | Provide information about survey anonymity and confidentiality and describe what mechanisms were used to protect unauthorized access. |  |
| Statistical  analysis | 10a | Describe statistical methods and analytical approach. Report the statistical software that was used for data analysis. |  |
|  | 10b | Report any modification of variables used in the analysis, along with reference (if available). |  |
|  | 10c | Report details about how missing data was handled. Include rate of missing items, missing data mechanism (i.e., missing completely at random [MCAR], missing at random [MAR] or missing not at random [MNAR]) and methods used to deal with missing data (e.g., multiple imputation). |  |
|  | 10d | State how non-response error was addressed. |  |
|  | 10e | For longitudinal surveys, state how loss to follow-up was addressed. |  |
|  | 10f | Indicate whether any methods such as weighting of items or propensity scores have been used to adjust for non-representativeness of the sample. |  |
|  | 10g | Describe any sensitivity analysis conducted. |  |
| **Results** | | |  |
| Respondent characteristics | 11a | Report numbers of individuals at each stage of the study. Consider using a flow diagram, if possible. |  |
|  | 11b | Provide reasons for non-participation at each stage, if possible. |  |
|  | 11c | Report response rate, present the definition of response rate or the formula used to calculate response rate. |  |
|  | 11d | Provide information to define how unique visitors are determined. Report number of unique visitors along with relevant proportions (e.g., view proportion, participation proportion, completion proportion). |  |
| Descriptive  results | 12 | Provide characteristics of study participants, as well as information on potential confounders and assessed outcomes. |  |
| Main findings | 13a | Give unadjusted estimates and, if applicable, confounder-adjusted estimates along with 95% confidence intervals and p-values. |  |
|  | 13b | For multivariable analysis, provide information on the model building process, model fit statistics, and model assumptions (as appropriate). |  |
|  | 13c | Provide details about any sensitivity analysis performed. If there are considerable amount of missing data, report sensitivity analyses comparing the results of complete cases with that of the imputed dataset (if possible). |  |
| **Discussion** | | |  |
| Limitations | 14 | Discuss the limitations of the study, considering sources of potential biases and imprecisions, such as non-representativeness of sample, study design, important uncontrolled confounders. |  |
| Interpretations | 15 | Give a cautious overall interpretation of results, based on potential biases and imprecisions and suggest areas for future research. |  |
| Generalizability | 16 | Discuss the external validity of the results. |  |
| **Other sections** | | |  |
| Role of funding source | 17 | State whether any funding organization has had any roles in the survey’s design, implementation, and analysis. |  |
| Conflict of interest | 18 | Declare any potential conflict of interest. |  |
| Acknowledgements | 19 | Provide names of organizations/persons that are acknowledged along with their contribution to the research. |  |
